# Classification of hallux valgus deformity–is there a standard?

**DOI:** 10.1007/s00402-024-05522-z

**Published:** 2024-09-11

**Authors:** Fabian T. Spindler, Sarah Ettinger, D. A. F. Scientific committee, Christina Stukenborg-Colsman, Sabine Ochman, Stefan Rammelt, Hans Polzer, Natalia Gutteck, Norbert Harrasser, Christian Plaass, Sebastian F. Baumbach

**Affiliations:** 1 Department of Orthopaedics and Trauma Surgery, Musculoskeletal University Center Munich (MUM), University Hospital, LMU Munich, Ziemssenstraße 5, 80336 Munich, Germany; 2 University Hospital for Orthopaedics and Trauma Surgery, Pius-Hospital Oldenburg, Georgstrasse 12, 26121 Oldenburg, Germany; 3 D.A.F. Scientific Committee: Deutsche Assoziation Für Fuß Und Sprunggelenk E.V, Strasse Des 17. Juni 106-108, 10623 Berlin, Germany

**Keywords:** Hallux valgus deformity, Classification, Intermetatarsalangle, Hallux valgus angle, Radiological

## Abstract

**Introduction:**

Hallux valgus deformity severity is one determent for the surgical procedure for hallux valgus (HV) correction. HV deformities are usually classified into mild/moderate/severe. The aim was to investigate the cut-off criteria used to classify HV deformity.

**Materials and Methods:**

The study was based on a previous living systematic review. Four common databases were searched for the last decade. All review-steps were conducted by two reviewers. Data assessed were the individual cut-off values used to classify HV deformity into mild/moderate/severe, and the referenced classification systems.

**Results:**

46 studies were included. 21/18 studies grade deformity based on the intermetatarsal angle (IMA)/ hallux valgus angle (HVA) with great heterogeneity throughout the different cut-off values. The most referenced classification systems were the Coughlin and Mann’s and the Robinson classification.

**Conclusions:**

The currently used classification systems are heterogenic, and no standard could be defined. The community should define a uniform classification system.

Level of Evidence.

Level I, systematic review of randomized controlled trials and prospective comparative studies.

## Introduction

More than 100 different surgical techniques have been published for correction of hallux valgus deformity [1, 2], with the severity of the hallux valgus deformity usually as the main determent for the surgical procedure [3, 4]. The degree of the deformity is commonly rated by the intermetatarsal angle (IMA) and the hallux valgus angle (HVA). Based on the combination of both, IMA and HVA, the deformity is frequently categorized into mild, moderate, or severe [1]. Up to now, the authors considered the classification to be consistently applied throughout the literature.

During the course of a living systematic review [5], initiated for the German hallux valgus guidelines, the authors became aware, that various cut-off criteria were used to classify hallux valgus severity. Still, as the degree of deformity is frequently considered as the predominant factor for choosing the surgical procedure, varying classifications will result in differing surgical approaches despite a comparable deformity. This subsequently leads to a considerable selection bias, which limits any comparative analysis of the literature available.

The aim of the current study was to investigate the cut-off criteria used to classify hallux valgus deformity into mild, moderate, or severe.

## Materials and methods

### Study selection

The study was based on a previous living systematic review [5] and was conducted per the Preferred Reporting Items for Systematic Reviews and Meta-Analyses (PRISMA-P) guidelines [6] and the PICOS criteria [7] and a priori registered (Prospero #CRD42021261490). Included were only prospective comparative studies comparing two surgical procedures or the same procedure for different degrees of deformity. Eligible studies must have reported at least one objective outcome parameter. Four common databases (MEDLINE (PubMed), Scopus, Central, and EMBASE) were searched from 01/01/2012 to 01/31/2023. The whole study selection-, level of evidence-, risk of bias-, and data extraction assessment was conducted by two reviewers independently (SE, SFB).

### Data assessed

The level of evidence was rated per the recommendations of Wright et al. [8] and the risk of bias was assessed by the Risk of Bias 2 (RoB 2) tool [9] or the Newcastle–Ottawa scale [10], where appropriate. The data assessed were the classification systems cited/used and the stated cut-off values for the IMA and HVA. In case the authors did not state on the actual cut-off values but reported a reference, the cut-off values of the respective reference were used.

### Statistics

Based on the final data sheet, the lower- (LB) and upper bounds (UB) for the categories mild (UB only), moderate (LB and UB) and severe (LB only) were analyzed. The analysis performed was descriptive, values are presented as mean ± SD, and were calculated using IBM Statistical Package for the Social Sciences, version 28 (SPSS).

## Results

### Study selection

**Fig. 1:**
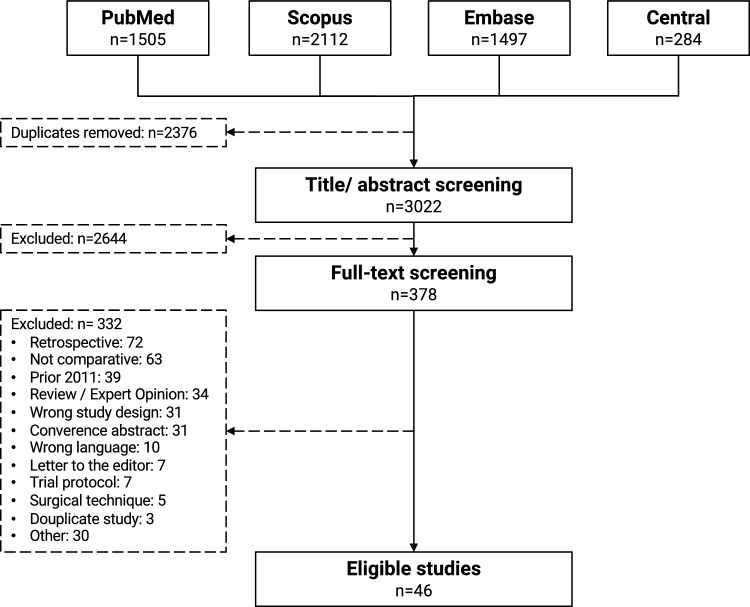
PRISMA flow chart

The study selection process is outlined in Fig. 1. 46 studies [11–56] were finally eligible for further analysis, including 30 RCTs (RoB2: 2 × high risk, 28 moderate risk) and 16 non-randomized comparative studies (Newcastle–Ottawa-Scale: 6 ± 1 points ≙ moderate risk).

### Data analysis

Out of the 46 studies included [11–56], any cut-off value for the IMA / HVA was stated in 21 studies (46%) [12, 14, 16–18, 21–24, 31, 34–36, 39, 42, 44, 45, 50, 52, 54, 55] / 18 studies (39%) [12, 14, 16, 17, 21, 31–36, 42, 44, 45, 47, 50, 54, 55]. Two studies were excluded due to missing cut-off values [41] or inconclusive data [43]. One paper [52] showed a discrepancy between the stated cut-off values and the values given in the associated reference. Subsequently the cut-off values of the cited paper were used. In one study [22] the referenced paper did not present any cut-off values. Therefore, the cut-off values stated in the paper were used.

The most commonly referenced classification systems were the Coughlin and Mann’s [57] (n = 5) [11, 12, 14, 17, 36] as well as the Robinson classification [58] (n = 4) [16, 31, 39, 52].

**Fig. 2:**
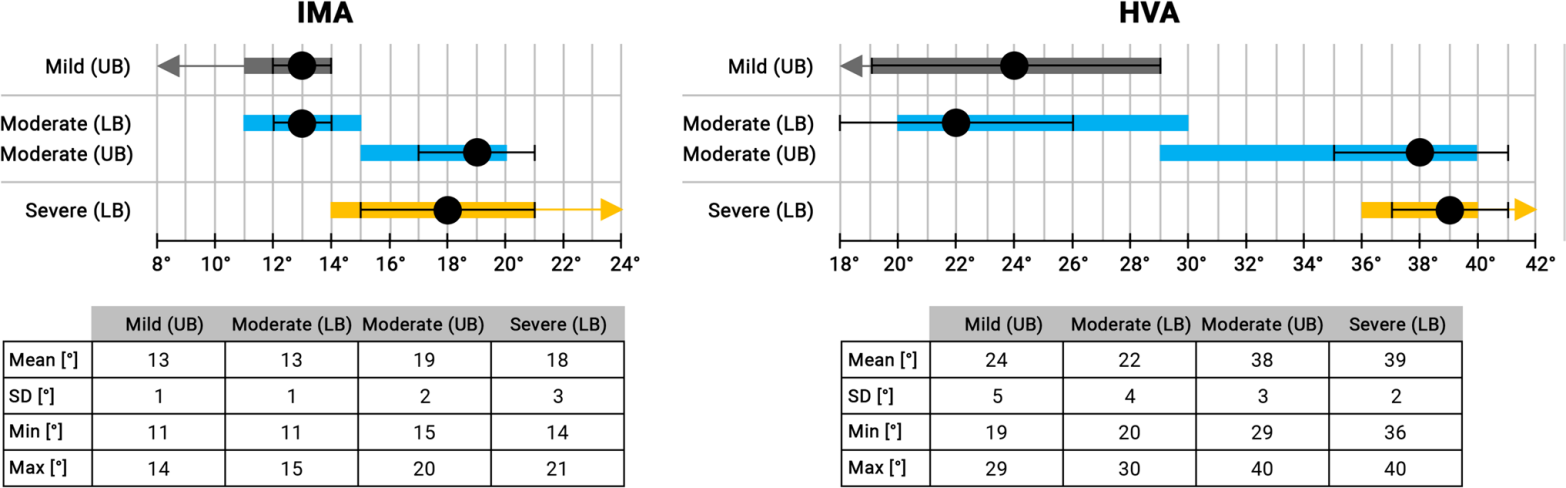
Cumulative analysis of the cut-off values for IMA and HVA used in literature.*UB* Upper bound, *LB* Lower bound, *SD* Standard deviation, *Min* Minimum, *Max* Maximum, *°* Degrees

Figure 2 depicts a cumulative analysis of the IMA and HVA values found in the studies included. Overall, a great heterogeneity was observed for the lower-(LB) and upper bound (UB) values applied in the literature, for both the IMA and HVA.

## Discussion

The analysis of the classification systems for grading the severity of the hallux valgus deformity used in literature revealed a tremendous heterogeneity for both the IMA and HVA.

To the best knowledge of the authors’ until now no study has investigated the different classification systems, i.e. cut-off values, used to rate the severity of a hallux valgus deformity. The current systematic review only included comparative, clinical outcome studies. As the choice of the surgical procedure is traditionally based on the degree of deformity [3, 4], their classification is of high relevance. The current systematic review revealed a considerable heterogeneity per the cut-off criteria for the different grades in the individual studies. For example, an IMA of 14° can be graded as mild, moderate, or severe, depending on the reference cited. Table 1 provides an overview of different classification systems published and a consensus on the data identified in the current study. The most referenced classification systems were those by Coughlin and Mann [57] and Robinson and Limbers [58]. Coughlin and Mann have just published the 10th volume [59]. Interestingly, their classification apparently has changed over time as well. The Robinson and Limbers classification has also been recommended in the Dutch national guidelines for hallux valgus (Federatie Medisch Specialisten, Richtlijnen Database; VS. July 29th 2021).

**Table 1:** Outline of different classification systems for hallux valgus deformity

	Guideline Germany (old Ver.) [57]		Guideline Netherland & Robinson 2005 [55]		Coughlin and Mann 2013 [54]		Current study*	
	IMA	HVA	IMA	HVA	IMA	HVA	IMA	HVA
Mild	< 16°	< 31°	< 14°	< 20°	< 11°	< 20°	< 13°	< 25°
Moderate	16°–20°	31°–40°	14°–20°	20°–40°	11°–16°	20°–40°	13°–18°	25–38°
Severe	> 20°	> 40°	> 20°	> 40°	> 16°	> 40°	> 18°	> 38°

*IMA*: Intermetatarsal angle, *HVA* Hallux valgus angle

^*^The values were extrapolated from the cumulative analysis in terms of a literature synopsis

In 2022, the American College of Foot and Ankle Surgeons® published a consensus statement on hallux valgus [60]. Overall, the consensus group could not reach a consensus on whether the “procedural selection for hallux valgus should be based on the severity of the deformity”. Amongst others, they argued that approaching evidence is pointing at the relevance of frontal plane deformity, i.e. pronatory rotation and hindfoot driven pronation. Therefore, traditional classifications, which are based on the transverse plane deformity, might not sufficiently characterize the deformity, and can therefore not indicate the necessary surgical procedure [61]. Furthermore, minimal invasive procedures have extended the deformity correction potential compared to traditional open osteotomies [4, 62–65]. With the approach of these novel diagnostic and treatment approaches, we might be in the need for novel classification systems. These should then be defined and applied uniformly throughout literature.

## Conclusion

Overall, the currently used classification systems are heterogenic. Therefore, any inter-study comparison is limited. Moreover, they probably underestimate the multidimensional nature of the deformity. With the approach of novel diagnostic tools, i.e. weightbearing CT, and treatment strategies, i.e. minimal invasive surgery, novel classifications must be developed [66]. But only their standardization throughout literature will allow a sufficient inter-study comparison and therefore generate the highest level of evidence.
